# Significant association of YAP1 and HSPC111 proteins with poor prognosis in Chinese gastric cancer patients

**DOI:** 10.18632/oncotarget.17932

**Published:** 2017-05-17

**Authors:** Shanshan Huang, Lingling Zhu, Yuan Cao, Li Li, Yongtao Xie, Jun Deng, Jianping Xiong

**Affiliations:** ^1^ Department of Oncology, The First Affiliated Hospital of Nanchang University, Nanchang, Jiangxi Province, 330006, China; ^2^ Medical College of Nanchang University, Nanchang, Jiangxi Province, 330006, China

**Keywords:** gastric cancer, YAP1, HSPC111, prognosis

## Abstract

Hippo-YAP1 is a tumor-suppressor signaling pathway that inhibits cell proliferation and accelerates apoptosis. However, the role of YAP1 in gastric cancer (GC) is still in dispute. Ribosomal biogenesis is closely correlated with human malignancies. HBV pre-S2 trans-regulated protein 3 (HSPC111) is a portion of an RNA-dependent complex and plays a crucial role in ribosome biosynthesis. Nevertheless, little is known about the expression and function of this factor in GC. In the present study, we evaluated the significance of YAP1 together with HSPC111 in gastric cancer. According to The Cancer Genome Atlas database, high YAP1 mRNA expression was significantly associated with poor prognosis of GC patients, and dramatically increased mRNA levels of HSPC111 are observed in GC tissues. Consistent with these findings, we detected increased expression of both YAP1 and HSPC111 in GC cell lines and clinical samples. Notably, nuclear expression of YAP1 was positively correlated with clinical stage (*P* = 0.041), tumor size (*P* = 0.023), and lymph node metastasis (*P* = 0.007), while HSPC111 expression was correlated with lymph node metastasis (*P* = 0.014). Our analyses also detected a correlation between HSPC111 expression and nuclear and cytoplasmic YAP1 in clinical samples (nuclear: *r* = 0.2615, *P* = 0.004; cytoplasm: *r* = −0.3721, *P* < 0.001) and cell lines. Finally, we showed that patients who were HSPC111- and nuclear YAP1-positive were associated with the worst prognosis (34.5 ± 4.8 months, *p* = 0.001), and that nuclear expression of YAP1 might act as an independent prognostic factor for GC patients.

## INTRODUCTION

Gastric cancer (GC), one of the most lethal malignancies worldwide, is the second leading cause of cancer-related death in China [1]. Due to a lack of specific symptoms, and the tendency of tumor invasion and metastasis, most patients are diagnosed with GC at an advanced stage. As a result, the 5-year survival rate of GC patients is typically less than 30% [2]. In The Cancer Genome Atlas (TCGA), GC is divided into four molecular subtypes: tumors positive for Epstein–Barr virus (9%), microsatellite unstable tumors (22%), genomically stable tumors (20%), and tumors with chromosomal instability (50%) [3]. The occurrence and progression of GC can be attributed to complex interactions between genetic, epigenetic, and environmental factors [4]. The poor prognosis of GC is primarily due to a limited understanding of its etiology and pathogenesis. As such, to improve the survival rate of GC patients, it is imperative to explore novel prognostic biomarkers and therapeutic targets.

Because many core pathways that regulate cell proliferation and survival are dysregulated in various tumors [5, 6], it is likely that abnormalities of these core-signaling pathways contribute to the development of GC. The Hippo-YAP1 pathway plays a crucial role in cell proliferation, differentiation, development, and apoptosis [7]. The core components of the Hippo pathway in mammals are MST1/2, WW45, LATS1/2, and Mob1. In the Hippo pathway, MST1/2 activation leads to phosphorylation and activation of LATS1/2. Activated LATS1/2, in turn, inhibits the transcriptional co-activators YAP and TAZ through phosphorylation [8–10]. Once phosphorylated, YAP/TAZ cannot accumulate in the nucleus and their co-transcriptional activity is hampered [11, 12]. Aberrant inactivation of this pathway leads to cell proliferation and promotes carcinogenesis. Overall, emerging evidence has shown that the Hippo pathway is strongly associated with several types of cancer [13–15], including GC [16, 17].

HBV pre-S2 trans-regulated protein 3 (HSPC111) is a nucleolar protein and a direct transcriptional target of c-Myc [18, 19]. HSPC111 comprises a portion of an RNA-dependent complex, depositing in the 40 to 80S region, and plays a role in ribosome biosynthesis [18]. HSPC111 also interacts with RNA 3′-phosphate cyclase, which catalyzes the transformation of a 3′-phosphate group into the 2′,3′-cyclic phosphodiester at the 3′ end of RNA [20, 21]. Down-regulation of HSPC111 and RNA 3′-phosphate cyclase dramatically hinders overall ribosomal (r)RNA synthesis and consequent protein translation in tumor cells [22]. Recently, increased HSPC111 expression was detected in breast cancer tissues and was found to correlate with adverse prognoses for breast cancer patients [18]. Furthermore, enhanced expression of HSPC111 was observed in prostate, liver, colorectal, pancreatic, and testicular cancer tissues [23–26].

In the present study, we investigated the expression and biological significance of YAP1 and HSPC111 in GC using data collected in the TCGA database, as well as clinical samples and cell lines. Our results demonstrate that expression of both YAP1 and HSPC111 were elevated in GC, and that the expression of these two factors was significantly correlated. Moreover, the expression of nuclear YAP1 was positively correlated with clinical stage, tumor size, and lymph node metastasis, while HSPC111 expression was significantly correlated with lymph node metastasis. Lastly, we show that the simultaneous expression of YAP1 and HSPC111 indicated the poorest prognosis for GC patients.

## RESULTS

### The importance of the Hippo-YAP1 pathway in GC based on TCGA database analysis

**Figure 1:**
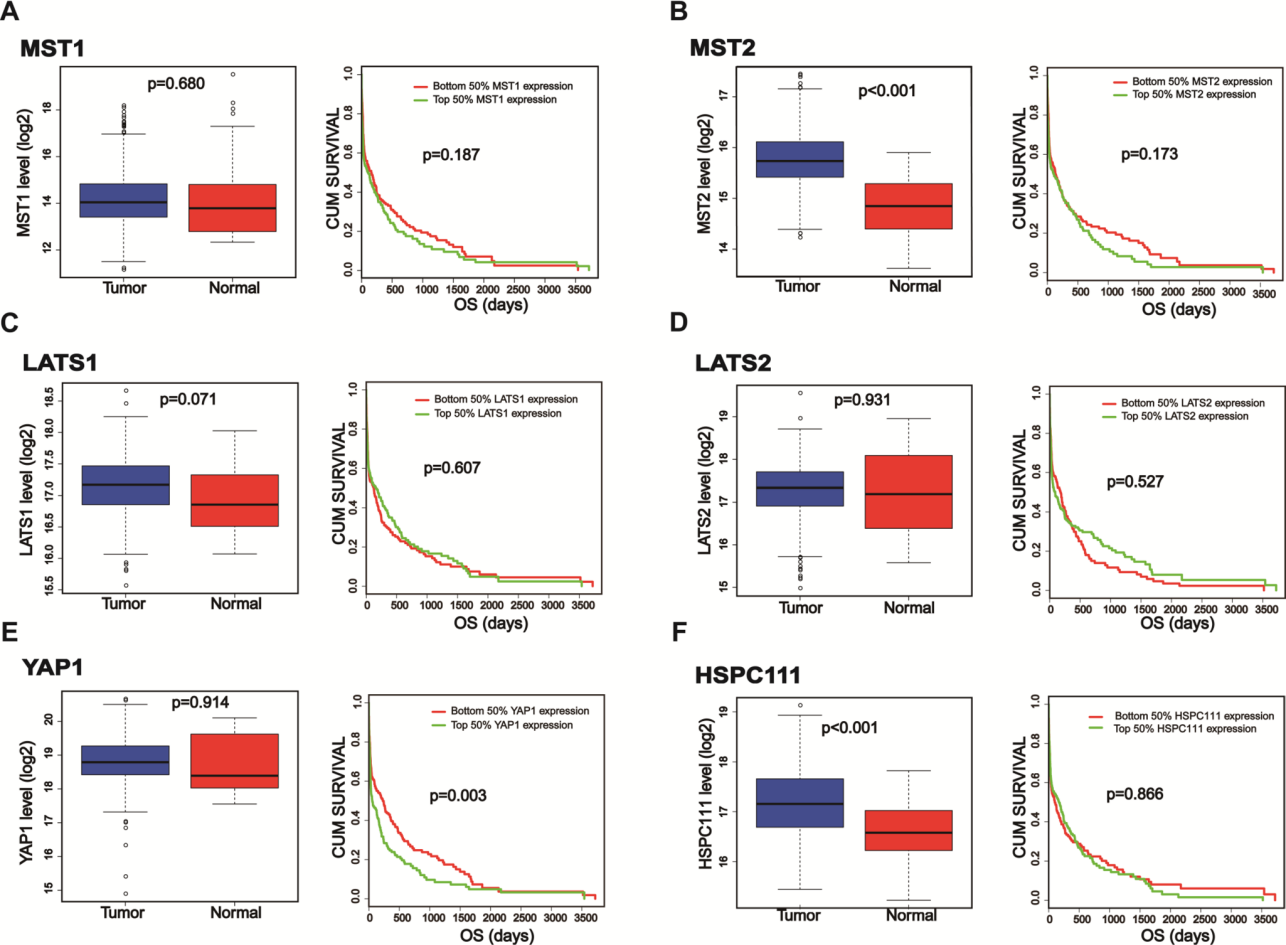
The expression levels as well as survival analysis comparisons of the core components of the Hippo-YAP1 pathway and HSPC111 TCGA database was searched, and the datasets of enrolled GC samples and related clinicopathologic information can be downloaded at https://gdc-portal.nci.nih.gov/projects/TCGA-STAD (**A**–**E**) The mRNA expression levels as well as survival analysis comparisons of the core components of the Hippo-YAP1 pathway in gastric cancer patients. (**F**) The mRNA expression level as well as survival analysis comparison of HSPC111 in gastric cancer patients.

Because the Hippo-YAP1 pathway is dysregulated in human GC [16, 17], we evaluated the expression levels of its core components and performed a survival analysis comparison using data collected in the TCGA database. The datasets of enrolled GC samples and related clinicopathologic information can be downloaded at https://gdc-portal.nci.nih.gov/projects/TCGA-STAD Though, we found no differences in the mRNA expression levels of MST1, LATS1, LATS2, or YAP1 between normal and GC tissues. In contrast, MST2 mRNA expression was higher in primary cancer samples than in normal samples. Meanwhile, survival analysis indicated that only high YAP1 mRNA expression was a predictor of poor prognoses for GC patients (Figure 1). These findings prompted us to further evaluate the role of YAP1 in GC.

### Expression of YAP1 and HSPC111 mRNA and protein in fresh tissues

**Figure 2:**
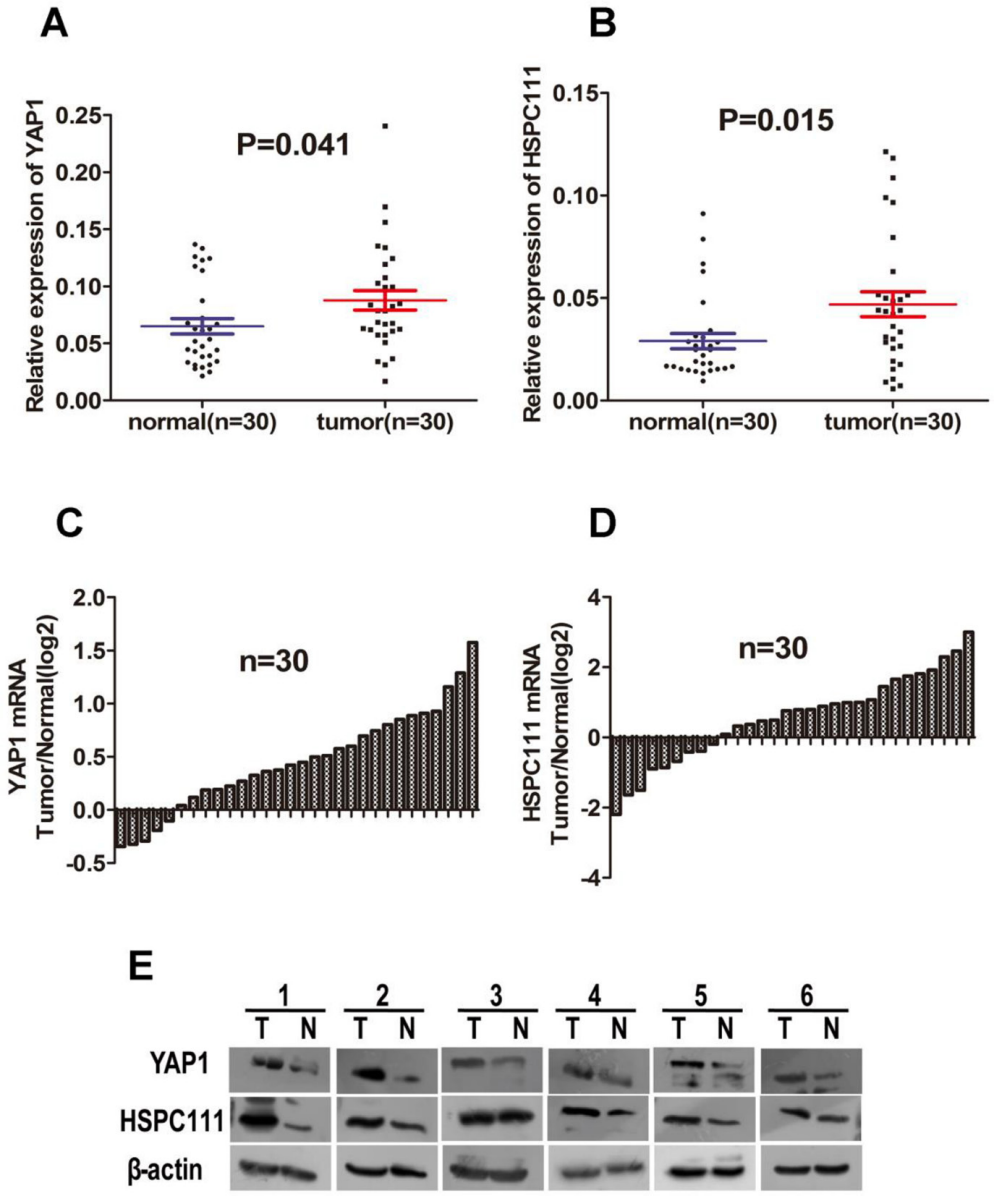
The mRNA and protein levels of YAP1 and HSPC111 in 30 paired gastric cancer (GC) samples (**A** and **B**) Scatter plots of the relative expression levels of (A) YAP1 and (B) HSPC111 mRNA in cancerous tissues and adjacent normal tissues. (**C** and **D**) Bar plots of (C) YAP1 and (D) HSPC111 expression in GC tissues, compared with paired normal tissues. (**E**) Representative protein expression levels of YAP1 and HSPC111 in six tumor (T) and matched normal (N) tissues. β-actin was used as an endogenous control.

YAP1 is a transcriptional co-activator in Hippo signaling and has received extensive attention for its remarkable biological properties in cancers [27, 28]. The target genes of YAP1 include connective tissue growth factor, CYR61, survivin, and c-Myc [29–31]. As stated above, HSPC111 is a direct transcriptional target of c-Myc. Because both YAP1 and HSPC111 are linked to c-Myc, we hypothesized that they could be valuable biomarkers of tumor progression, and that the YAP1 and HSPC111 co-expression status might hold significance for GC. To test this hypothesis, we first compared the expression level of HSPC111 between GC tissues and adjacent normal gastric tissues, based on information provided in the TCGA database (Figure 1F). Notably, the mRNA levels of HSPC111 are dramatically increased in GC tissues. Next, we examined the expression patterns of YAP1 and HSPC111, and their relationship, in GC tissues. For these experiments, a cohort of 30 fresh-frozen GC samples and corresponding normal gastric tissues were subjected to western blot and quantitative real-time polymerase chain reaction (qRT-PCR) analyses. As shown in Figure 2A and 2B, the mRNA levels of YAP1 and HSPC111 were significantly higher in GC tissues than in the matched normal tissues (*P* = 0.041 and *P* = 0.015, respectively). When we defined a greater than 1-fold change in mRNA expression as up-regulation and less than this as down-regulation, 86.7% (26/30) and 70% (21/30) of the GC tissues examined exhibited up-regulation of YAP1 and HSPC111, respectively (Figure 2C and 2D). Western blot analysis verified that the protein levels of YAP1 and HSPC111 were also markedly higher in GC tissues than in the corresponding normal tissues (Figure 2E). Together, these results indicate that YAP1 and HSPC111 are up-regulated at both the mRNA and protein levels in human GC.

### Immunohistochemistry for YAP1 and HSPC111

**Figure 3:**
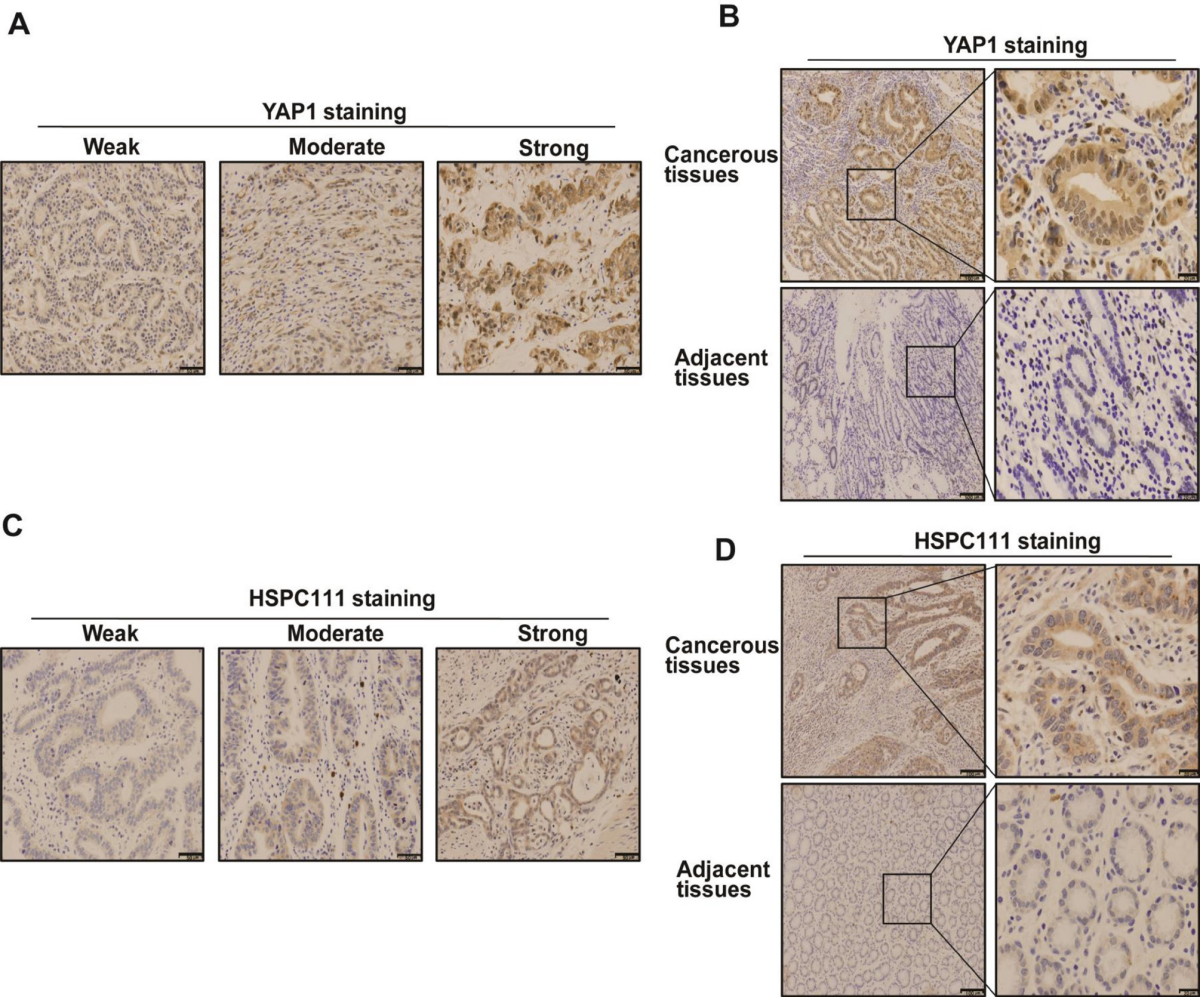
Immunohistochemical staining pattern of YAP1 and HSPC111 in gastric cancer (GC) tissues (**A** and **C**) The expression pattern of YAP1 and HSPC111, based on percentage and intensity of stained cells, in GC tissues. (**B** and **D**) Representative images of YAP1- and HSPC111-positive/negative staining in GC tissues and adjacent normal tissues.

To confirm the results obtained by qRT-PCR and western blot analyses, expression of YAP1 and HSPC111 was examined in 120 GC and 30 normal gastric tissue samples by immunohistochemistry. Staining of these proteins ranged from weak to strong (Figure 3A and 3C). YAP1-positive staining was confined primarily to the cytoplasm and nuclei of tumor cells, while HSPC111-positive staining was confined mainly to the cytoplasm of tumor cells. Notably, there were obvious differences in the expression levels of both proteins between cancerous and adjacent normal tissues (Figure 3B and 3D); the positive expression rate of YAP1 (86.7%, 104/120) and HSPC111 (40%, 48/120) in GC samples was significantly higher than that in normal gastric samples (66.7%, 20/30 and 16.7%, 5/30; *P* = 0.015 and *P* = 0.019, respectively).

As reported, YAP1 was expressed in both the cytoplasm and nuclei of GC cells [32]. Therefore, we assessed whether its subcellular location could contribute to the discrepancy in positivity between GC and normal tissues. Only 26.7% (8/30) of the YAP1 nuclei stained in normal gastric tissues were positive, while 48.3% (58/120) were positive in GC tissues (*P* = 0.032). Interestingly, we did not observe any differences in cytoplasmic staining of YAP1 in GC tissues (76.7%, 92/120) versus normal gastric tissues (63.3%, 19/30; *P* = 0.136).

**Figure 4:**
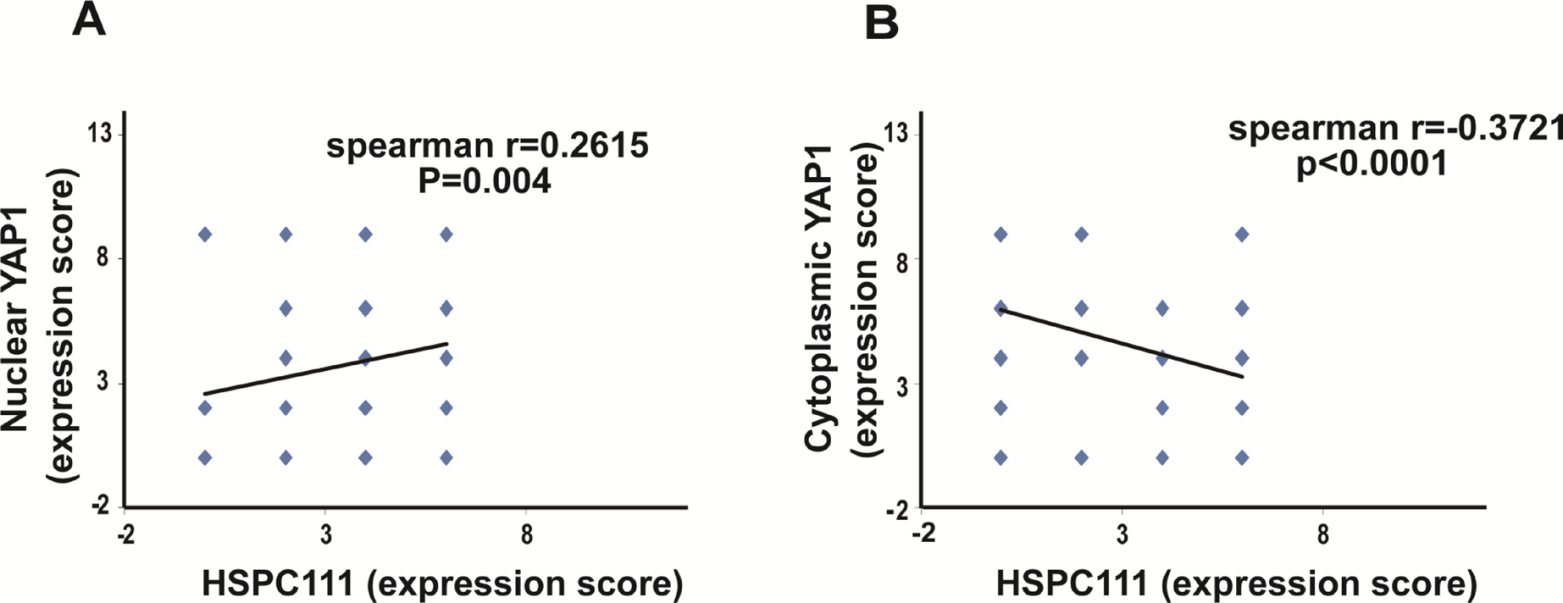
Correlation between YAP1 and HSPC111 expression in gastric cancer samples (**A**) Correlation between nuclear YAP1 expression and HSPC111 expression. (**B**) Correlation between cytoplasmic YAP1 expression and HSPC111 expression.

Notably, Spearman correlation coefficient analysis revealed that HSPC111 levels were positively correlated with nuclear YAP1 (*r* = 0.2615, *P* = 0.004; Figure 4A), but negatively correlated with cytoplasmic YAP1 (*r* = −0.3721, *P* < 0.001; Figure 4B) expression. Overall, nuclear expression of YAP1 and cytoplasmic expression of HSPC111 were up-regulated in GC tissues, and cytoplasmic HSPC111 expression correlated with both nuclear and cytoplasmic YAP1 levels.

### Expression of YAP1 and HSPC111 and their relationships to the clinical characteristics of GC

To determine the clinical significance of YAP1 and HSPC111 expression in GC, the chi-square test was adopted to examine their relationships with regard to gender, age, histological differentiation, tumor size, location of primary tumor, depth of invasion, lymph node metastasis, and clinical stage (Table 1). The expression levels of nuclear YAP1 and HSPC111 were both significantly related to lymph node metastasis (*P* = 0.007 and *P* = 0.014, respectively). Moreover, elevated nuclear YAP1 expression was significantly associated with clinical stage (*P* = 0.041) and tumor size (*P* = 0.023). No significant correlations between HSPC111 or nuclear YAP1 expression and other clinicopathological parameters were detected.

**Table 1:** Relationship between HSPC111 and nuclear YAP1 expression and clinicopathological variables (*n* – 120)

Variables	Number	YAP1 expression		*P* value	HSPC111 expression		*P* value
		Positive	Negative		Positive	Negative	
**Gender**				0.698			0.617
Male	87	43 (49.4)	44 (50.6)		36 (41.4)	51 (58.6)	
Female	33	15 (45.45)	18 (54.5)		12 (36.4)	21 (63.6)	
Age*				0.273			0.456
> 57.5	60	32 (53.3)	28 (46.7)		22 (36.7)	38 (63.3)	
< 57.5	60	26 (43.3)	34 (56.7)		26 (43.3)	34 (56.7)	
**Depth of invasion**				0.353			0.437
T1/2	38	16 (42.1)	22 (57.9)		13 (34.2)	25 (65.7)	
T3/4	82	42 (51.2)	40 (48.8)		35 (42.7)	47 (57.3)	
**LNM**				**0.007**			**0.014**
Yes	46	15 (32.6)	31 (67.4)		12 (26.1)	34 (73.9)	
No	74	43 (58.1)	31 (41.9)		36 (48.6)	38 (51.4)	
**Tumor size**				**0.023**			0.184
< 6 cm	101	48 (47.5)	53 (52.5)		43 (42.6)	58 (57.4)	
> = 6 cm	19	10 (52.6)	9 (47.4)		5 (26.3)	14 (73.7)	
**Differentiation**				0.298			0.057
Low/undifferentiated	86	39 (45.3)	47 (54.7)		39 (45.3)	47 (54.7)	
High/moderate	34	19 (55.9)	15 (44.1)		9 (26.5)	25 (73.5)	
**TNM stage**				**0.041**			0.454
I/II	55	21 (38.2)	34 (61.8)		20 (36.4)	35 (63.6)	
III	65	37 (56.9)	28 (43.1)		28 (43.1)	37 (56.9)	

Age* = mean age; LNM = lymph node metastasis.

We next investigated the relationship between the combined expression of HSPC111 and nuclear YAP1 and the clinicopathological features of GC patients. The group with increased expression of both HSPC111 and nuclear YAP1 showed poorer differentiation than the other groups (Table 2), suggesting that HSPC111+/nuclear YAP1+ GC patients manifested higher-grade malignancy.

**Table 2:** HSPC111/nuclear YAP1 expression and clinicopathological variables in GC patients

		HSPC111 (+)/ Nuclear YAP1 (+)	HSPC111 (+)/ Nuclear YAP1 (−)	HSPC111 (−)/ Nuclear YAP1 (+)	HSPC111 (−)/ Nuclear YAP1 (−)	*P* value
Variables	Number	*n* (29)	*n* (19)	*n* (29)	*n* (43)	
**Gender**						0.828
Male	86	21	15	21	29	
Female	34	8	4	8	14	
Age*						0.291
>57.5	60	13	9	19	19	
<57.5	60	16	10	10	24	
**Depth of invasion**						0.232
T1/2	38	5	8	11	14	
T3/4	82	24	11	18	29	
**LNM**						**0.035**
Yes	48	7	7	12	22	
No	72	22	12	17	21	
Tumor size						0.127
< 6cm	101	23	19	22	37	
> = 6cm	19	6	0	7	6	
**Differentiation**						0.126
Low/undifferentiated	86	22	17	17	30	
High/moderate	34	7	2	12	13	
**TNM stage**						0.376
I/II	55	10	10	12	23	
III	65	19	9	17	20	

(*n* = 120).

Age* = mean age; LNM = lymph node metastasis.

### Prognostic value of YAP1 and HSPC111 expression

**Figure 5:**
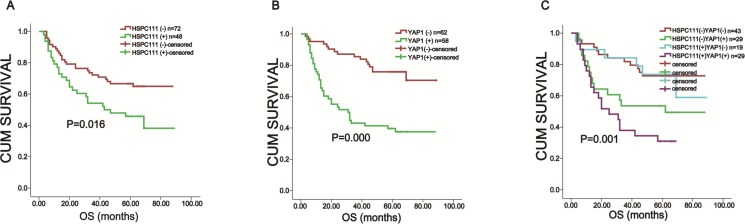
Kaplan-Meier survival analysis based on YAP1 and HSPC111 expression in gastric cancer (**A**) The overall survival of nuclear YAP1+ and nuclear YAP1- patients. (**B**) The overall survival of HSPC111+ and HSPC111- patients. (**C**) The overall survival of YAP1+/HSPC111+ patients.

Kaplan-Meier analysis and log-rank testing were used to evaluate the prognostic value of YAP1 and HSPC111 expression in GC. Patients who were HSPC111+ had worse overall survival (49.8 ± 5.2 months) rates than those who were HSPC111- (64.9 ± 3.9 months; *P* = 0.016) (Figure 5A). Likewise, positive nuclear YAP1 expression was associated with poorer overall survival than negative nuclear YAP1 expression (44.3 ± 4.7 months versus 72.9 ± 3.6 months, *P* = 0.000) (Figure 5B). Furthermore, nuclear YAP1+ patients exhibited shorter overall survival times (44.3 ± 4.7 months) than cytoplasmic YAP1+ patients (63.4 ± 3.5 months).

We further investigated the prognostic value of combined HSPC111 and nuclear YAP1 expression. Patients were classified into four groups: HSPC111+/nuclear YAP1+ (*n* = 29), HSPC111-/nuclear YAP1- (*n* = 43), HSPC111+/nuclear YAP1- (*n* = 19), and HSPC111-/nuclear YAP1+ (*n* = 29). Kaplan-Meier analysis showed that the HSPC111-/nuclear YAP1- group had the most favorable prognosis (70.8 ± 4.4 months), while the HSPC111+/nuclear YAP1+ group had the worst prognosis (34.5 ± 4.8 months; *P* = 0.001) (Figure 5C). These data indicate that combined expression of HSPC111 and nuclear YAP1 was a better survival biomarker than either alone.

Lastly, univariate and multivariate analyses were utilized to determine the independent prognostic factors of GC patients (Table 3). Univariate Cox regression analysis showed that depth of invasion (*P* = 0.000), lymph node metastasis (*P* = 0.000), clinical TNM stage (*P* = 0.000), tumor size (*P* = 0.003), HSPC111 expression (*P* = 0.019), nuclear YAP1 expression (*P* = 0.000) and HSPC111/nuclear YAP1 expression (*P* = 0.000) were significantly correlated with overall survival in GC patients. These factors were then subjected to multivariate Cox regression analysis, which indicated that depth of invasion (*P* = 0.014), tumor size (*P* = 0.010) and nuclear YAP1 expression (*P* = 0.000) were independent prognostic factors.

**Table 3:** Univariate and multivariate analysis of the correlation between clinicopathological parameters and prognostic significance of GC patients

Variables	Univariate analysis	*P* value	Multivariate analysis	*P* value
	HR (95% CI)		HR (95% CI)	
Sex(male vs. female)	1.847 (0.926–3.684)	0.082		
Age* (> 57.5 vs. < 57.5)	1.495 (0.862–2.594)	0.152		
Depth of invasion (T1/T2 vs. T3/T4)	0.122 (0.044–0.338)	**0.000**	0.221 (0.066–0.737)	**0.014**
LNM (Yes vs. No)	7.203 (3.066–16.923)	**0.000**	3.717 (0.862–16.027)	**0.078**
Tumor size (< 6 cm vs. > = 6 cm)	0.391 (0.208–0.734)	**0.003**	0.390 (0.190–0.802)	**0.010**
Differentiation(Low/undifferentiate vs. High/moderate)	0.784 (0.439–1.401)	0.412		
TNM stage (I/II vs. III)	0.158 (0.077–0.325)	**0.000**	0.863 (0.224–3.320)	0.830
HSPC111 (positive vs. negative)	1.925 (1.116–3.319)	**0.019**	2.532 (0.843–7.603)	0.098
Nuclear YAP1 (positive vs. negative)	3.458 (1.912–6.252)	**0.000**	5.113 (2.123–12.316)	**0.000**
HSPC111/Nuclear YAP1 (HSPC111+/Nuclear YAP1+ vs. all others)	3.128 (1.784–5.483)	**0.000**	0.483 (0.130–1.792)	0.277

Age* = mean age; LNM = lymph node metastasis.

### Correlation between YAP1 and HSPC111 in GC cell lines

**Figure 6:**
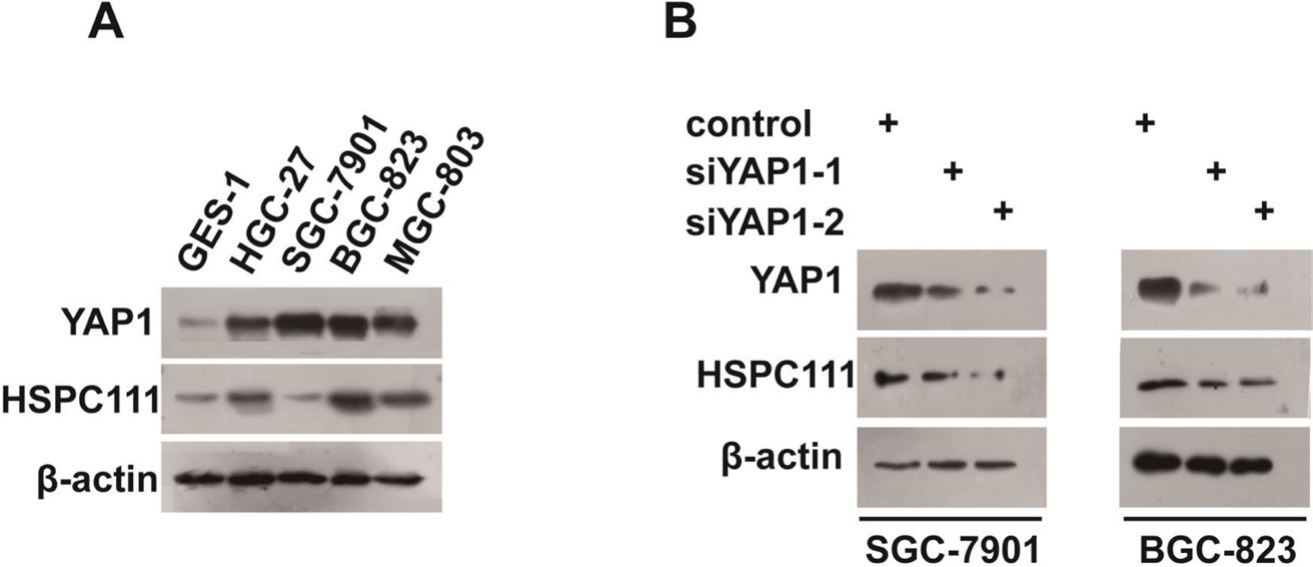
Correlation between YAP1 and HSPC111 expression in gastric cancer (GC) cell lines (**A**) Protein expression levels of YAP1 and HSPC111 in human gastric mucosal epithelial (GES1) and human GC cells lines. (**B**) Protein expression levels of HSPC111 upon YAP1 deletion in HGC-27 and BGC-823 cells.

To further confirm the correlation between YAP1 and HSPC111 expression in GC, we measured their expression in human gastric mucosal epithelial (GES1) and human GC cells. Both YAP1 and HSPC111 were up-regulated in cancer cell lines, compared with GES1 cells (Figure 6A). Moreover, knockdown of YAP1 expression contributed to the down-regulation of the level of HSPC111 (Figure 6B). Overall, our data indicate that expression of YAP1 and HSPC111 was significantly correlated.

## DISCUSSION

In the present study, we determined that both the protein and mRNA levels of YAP1 and HSPC111 were higher in most GC tissues. Additionally, we found that YAP1 expression correlated with lymph node metastasis, clinical stage, and tumor size, while HSPC111 expression correlated with lymph node metastasis alone. Because GC patients with positive expression of YAP1 and HSPC111 were associated with a poorer prognosis than those that were negative for YAP1 and HSPC111, we propose that these factors comprise prognostic biomarkers for GC patient outcomes. Spearman rank correlation analysis revealed a positive correlation between the expression of YAP1 and HSPC111. Furthermore, nuclear YAP1 expression status was an independent prognostic indicator for GC patients.

Hippo-YAP1 is a tumor-suppressor signaling pathway that inhibits cell proliferation and accelerates apoptosis. Dysregulation of this pathway is closely correlated with the initiation, development, and progression of GC [16]. As reported previously, compared with their expression in adjacent normal gastric tissues, MST1/2 and LATS1, which are upstream of the Hippo pathway, are usually down-regulated in GC tissues [33, 34]. However, TCGA database analyses indicated no significant difference between tumor and normal gastric tissues in the expression levels of MST1, LATS1, or LATS2. Conversely, MST2 expression appeared to be up-regulated in tumor tissues. One reasonable interpretation of this discrepancy is that our bioinformatics analysis reflected the mRNA expression levels of these genes, while previous reports utilized protein expression levels. However, the mechanism underlying the discrepancy in mRNA and protein expression levels of these genes requires further investigation.

YAP, the core downstream effector of the Hippo pathway, was first characterized in *Drosophila* (Yorkie) and is considered to be a potent oncoprotein [35]. Indeed, YAP1 is overexpressed in several human cancers and tumorigenic models [36, 37]. In the current study, we observed that YAP1 was overexpressed in GC tissues, which was consistent with the results of previous studies [32, 38–40]. Our data also revealed that nuclear YAP1 expression was closely correlated with poor prognosis, lymph node metastasis, clinical stage, and tumor size, suggesting that positive expression of YAP1 was an indicator of highly malignant GC. Interestingly, cytoplasmic YAP1 expression showed no significant correlation with the prognosis and clinicopathological parameters of GC patients. This discrepancy could potentially be due to the fact that YAP1 acts as a transcriptional co-activator of the Hippo pathway. In the presence of defects in Hippo signaling, or other stimuli, YAP1 translocates to the nucleus and binds to transcription factors (e.g., TEAD1-4, ErbB4, SMAD, and p73) to promote expression of genes that accelerate cell proliferation and hinder apoptosis [30, 41, 42]. As such, nuclear localization of this factor is likely necessary to mediate cellular effects. This transcriptional co-activator function of YAP1 also partly explains the poor prognostic value of this marker for overall survival.

Ribosomal biogenesis is vital for cell growth and proliferation, and increased ribosome production accelerates cell proliferation and leads to tumor evolution and progression [43, 44]. As was reported previously, enhanced rRNA synthesis is closely correlated with cell conversion and multiplication, and some rRNAs are overexpressed in malignant tumors [45, 46]. Studies have demonstrated that HSPC111 is a ribosomal protein located in a large RNA-dependent nucleolar complex, and plays an important role in regulating rRNA synthesis and ribosomal biogenesis [22, 47]. From these results, we can speculate that HSPC111 is crucially involved in tumor progression.

In the present study, we provided the first evidence that HSPC111 is up-regulated in GC tissues. Our data also revealed that HSPC111 expression correlated with vascular invasion and predicted poor outcomes for GC patients. Together, these results highlight a critical role of HSPC111 in GC. Interestingly, HSPC111 staining was detected in the cytoplasm of both GC and normal tissues; however, nuclear staining was faint or undetectable. One reasonable explanation for the cytoplasmic location of HSPC111 is that proteins associated with ribosomal biogenesis can shift from the nucleus to the cytoplasm. In addition, HSPC111 might play varying roles in different malignancies.

We speculated that HSPC111 might be a multifunctional protein involved in GC progression. In the nucleus, it might physically and functionally interact with certain ribosomal proteins, such as RPL5, RPL11, RPS10, and RPS17, and thereby competitively inhibit the ribosomal protein-MDM2 interaction and alleviate ribosomal protein-mediated suppression of MDM2 ubiquitin ligase activity toward p53 [48, 49]. In the cytoplasm, HSPC111 might directly interact with and stabilize MDM2, which could promote P53 degradation via ubiquitination and subsequently accelerate tumor progression [50]. Certainly, these hypotheses need to be further verified and validated.

Because HSPC111 is a direct transcriptional target of c-Myc [18, 19], and c-Myc is a downstream effector of YAP [31], we assessed the correlation between YAP1 and HSPC111 expression, and the prognostic value of their co-expression status in GC patients. Our findings revealed that the expression levels of YAP1 and HSPC111 were indeed positively correlated, and that nuclear YAP1+/HSPC111+ patients were associated with the poorest prognosis. It is reasonable to believe that there is a link between YAP1 and HSPC111 in GC, and there may be a synergistic effect between these factors on GC progression.

In conclusion, the results of our study reveal that expression of YAP1 and HSPC111 are up-regulated in GC. In addition, our data show that elevated expression of YAP1 and HSPC111 is negatively associated with the GC patient prognoses. Statistical analysis demonstrated that an increase in the expression of these two proteins was associated with the poorest survival rate among GC patients. Our study has revealed strong correlation between YAP1 and HSPC111 and is the first to suggest that the Hippo-YAP pathway might be implicated in ribosomal biogenesis.

## MATERIALS AND METHODS

### Patients and tissue specimens

Formalin-fixed, paraffin-embedded samples from 120 GC patients were collected for immunohistochemistry analysis at the Department of Pathology of the First Affiliated Hospital of Nanchang University, from January 2009 to December 2012. The clinicopathological features of the patients are provided in Table 1. Tumor clinical stage was evaluated according to the 2010 criteria of The American Joint Committee on Cancer. The 120 patients were followed for survival analysis until December 2016. Thirty paired tissue specimens from fresh GC and matched noncancerous tissues were frozen at −80°C until subjected to protein and RNA extraction for western blot and qRT-PCR analysis, respectively. None of the patients received chemotherapy or radiotherapy before surgery. All tissue specimens were collected with patient consent and the research was approved by the ethical committee of the First Affiliated Hospital of Nanchang University.

### Immunohistochemistry

Formalin-fixed, paraffin-embedded (4-μm-thick tissue) sections were used for immunohistochemical staining. Sections were incubated at 63°C overnight, deparaffinized with xylene, and rehydrated in graded ethanol to distilled water, then immersed in boiling ethylene diamine tetraacetic acid for 20 min for antigen retrieval. After antigen retrieval, slides were incubated in methanol containing 3% hydrogen peroxide to quench endogenous peroxidase activity. Next, the slides were incubated with primary antibodies specific to YAP1 (1:50) (Abcam, Cambridge, UK) and HSPC111 (1:100) (Abcam) at room temperature for 2 h. After washing with phosphate-buffered saline (PBS), the slides were treated with a secondary antibody at room temperature for 20 min. 3,3′-Diaminobenzidine tetrahydrochloride (Fuzhou Maixin Biological Technology, Fujian, China) was then added to detect antigen-antibody complexes. Finally, the slides were counterstained with Mayer's hematoxylin, air-dried, and mounted. Slides were evaluated by two independent pathologists; YAP1 and HSPC111 expression levels were scored according to the proportion of stained tumor cells and the intensity of the staining [51]. The proportion was scored as: 0 (0%), 1 (0–10%), 2 (10–50%), and 3 (50–100%). The intensity of staining was scored as: 0 (negative), 1 (weak), 2 (moderate), and 3 (strong). The final YAP1 and HSPC111 expression scores were calculated by multiplying the above two scores. Patients with a final score of < 4 were considered as negative expression.

### Western blot analysis

Total proteins were extracted from cells and the 30 pairs of frozen tissue specimens using RIPA lysis buffer (Applygen, Beijing, China). Protein lysates were separated on 12% sodium dodecyl sulfate polyacrylamide gels and transferred to polyvinylidene fluoride membranes using the semi-dry technique. After blocking with 5% nonfat milk in TBST (tris-buffered saline with Tween-20) for 1 h at room temperature, membranes were incubated with specific antibodies for HSPC111 (1:1,000) (Abcam) and β-actin (1:1,000) (Abcam) at 4°C overnight. After washing three times for 5 min with TBST, the membranes were incubated with the corresponding horseradish peroxidase-conjugated secondary antibodies at room temperature for 1 h. The membranes were then washed three times for 5 min with TBST and the target proteins were stained using an EasySee Western Blot Kit (TransGen Biotech, Beijing, China). Each experiment was repeated in triplicate.

### RNA extraction and quantitative real-time polymerase chain reaction

Total RNA was extracted from cells and the 30 pairs of frozen tissue specimens using Trizol reagent (Invitrogen, Carlsbad, CA, USA). cDNA was synthesized using the TransScript All-in-One First-Strand cDNA Synthesis kit (TransGen Biotech), according to the manufacturer's protocol. mRNA levels were determined with the Fast Start Universal SYBR Green Master mix (Takara Bio, Shiga, Japan). Glyceraldehyde 3-phosphate dehydrogenase (GAPDH) mRNA was used as the internal control. Primers were as follows:

HSPC111; 5′-GCGTCTGAACCGGAATGCTC-3′ (forward) and 5′-CCAGGTTCTGCCGTACCGAT-3′ (reverse); YAP1: 5′-CAGGAGCCCTGACTCCACAG-3′ (forward) and 5′-TTGCCATCTCCCAACCTGCT-3′ (reverse); GAPDH: 5′-CAGGGCTGCTTTTAACTCTGG T-3′ (forward) and 5′-GATTTTGGAGGGATCTCGCT-3′ (reverse). Amplification was conducted under the following conditions: denaturation at 95°C for 10 min, followed by 40 cycles of 95°C for 15 s and 60°C for 30 s. Experiments were performed in triplicate. Relative expression levels of HSPC111 were calculated using the 2^−ΔΔCT^ method.

### Cell culture and small interfering (si)RNAs

GES-1, BGC-823, HGC-27, SGC-7901, and MGC803 cells were purchased from the Shanghai Institute for Life Science, Chinese Academy of Sciences (Shanghai, China). All cell lines were cultured in RPMI1640 medium (Thermo Fisher Scientific, Waltham, MA, USA) supplemented with 10% fetal bovine serum (Thermo Fisher Scientific) and incubated at 37°C in a humidified incubator containing 5% CO_2_. Two siRNAs (YAP1siR-1, 5′-CUGCCACCAAGCUAGAUAATT-3′; YAP1siR-2, 5′-GGUGAUAUAUCAACCAAATT-3′) and their negative siRNA non-targeting control (5′-UUCUCCGAACGUGUCACGUTT-3′) were purchased from Genepharma (Shanghai, China).

### Statistical analyses

Chi-square or Fisher's exact tests were used to evaluate the relationship between clinicopathologic parameters and the protein expression levels of YAP1 and HSPC111. Univariate survival analysis and overall survival curves were plotted using the Kaplan-Meier method. Significant differences between these curves were analyzed via the log-rank test. The Cox proportional hazard model was adopted for multivariate analysis of the independent prognostic indicators for overall survival. The Spearman rank correlation model was used to analyze the relationship between the protein expression levels of YAP1 and HSPC111. Differences in the mRNA expression levels of YAP1 and HSPC111 in fresh-frozen GC and matched normal tissues were analyzed using paired *t*-tests. All analyses were performed using SPSS 18.0 software (SPSS Statistics, Inc., Chicago, IL, USA). All statistical tests were two sided. *P* < 0.05 was considered statistically significant.
